# Map-Matching-Based Localization Using Camera and Low-Cost GPS for Lane-Level Accuracy †

**DOI:** 10.3390/s22072434

**Published:** 2022-03-22

**Authors:** Rahmad Sadli, Mohamed Afkir, Abdenour Hadid, Atika Rivenq, Abdelmalik Taleb-Ahmed

**Affiliations:** 1 Institut d’Électronique de Microélectronique et de Nanotechnologie (IEMN), UMR 8520, Université Polytechnique Hauts de France, University of Lille, CNRS, Centrale Lille, F-59313 Valenciennes, France; atika.menhaj@uphf.fr (A.R.); abdelmalik.taleb-ahmed@uphf.fr (A.T.-A.); 2 Transalley Technopole, 59300 Famars, France; mohamed.afkir@transalley.com

**Keywords:** autonomous driving, lane-level localization, lane detection, GNSS, GPS, map-matching

## Abstract

For self-driving systems or autonomous vehicles (AVs), accurate lane-level localization is a important for performing complex driving maneuvers. Classical GNSS-based methods are usually not accurate enough to have lane-level localization to support the AV’s maneuvers. LiDAR-based localization can provide accurate localization. However, the price of LiDARs is still one of the big issues preventing this kind of solution from becoming wide-spread commodity. Therefore, in this work, we propose a low-cost solution for lane-level localization using a vision-based system and a low-cost GPS to achieve high precision lane-level localization. Experiments in real-world and real-time demonstrate that the proposed method achieves good lane-level localization accuracy, outperforming solutions based on only GPS.

## 1. Introduction

Autonomous driving or drive-less car system is a promising technology for the future transportation that potentially has the capacity to improve road safety and to have a better mobility. Self-driving cars promise to bring a number of benefits to society, including prevention of road accidents, optimal fuel usage, comfort and convenience [1].

An autonomous vehicle is a car that can drive itself without human intervention. It is therefore obvious that for this type of vehicle, it must be able to know its location very precisely and very quickly in order to know where it is on the road. Indeed, with such a steering system, the driving of an autonomous vehicle is automated. This system must enable it to travel and make decisions without the driver’s intervention.

In other words, an autonomous vehicle must be able to navigate independently without human intervention to reach a predetermined destination from the current location. When it comes to navigation task, it requires the understanding of situation of the surrounding environment and the knowing of its current location, which is the actual position relative to a predefined path toward the destination.

Determining the actual vehicle position, known as the vehicle localization, is a crucial task in autonomous car. An autonomous car must be able to localize precisely its position in order to perform various maneuvers such as changing lanes and turning. As humans, we can drive much faster and safer if we are familiar with the route or if we have been provided sufficient road signs and obstacles to guide us in driving on unfamiliar roads. We already know what to expect for the upcoming situations and conditions such as where the intersections and stop signs are, where and when to take a turn, turn left or turn right, and etc.

**Figure 1:**
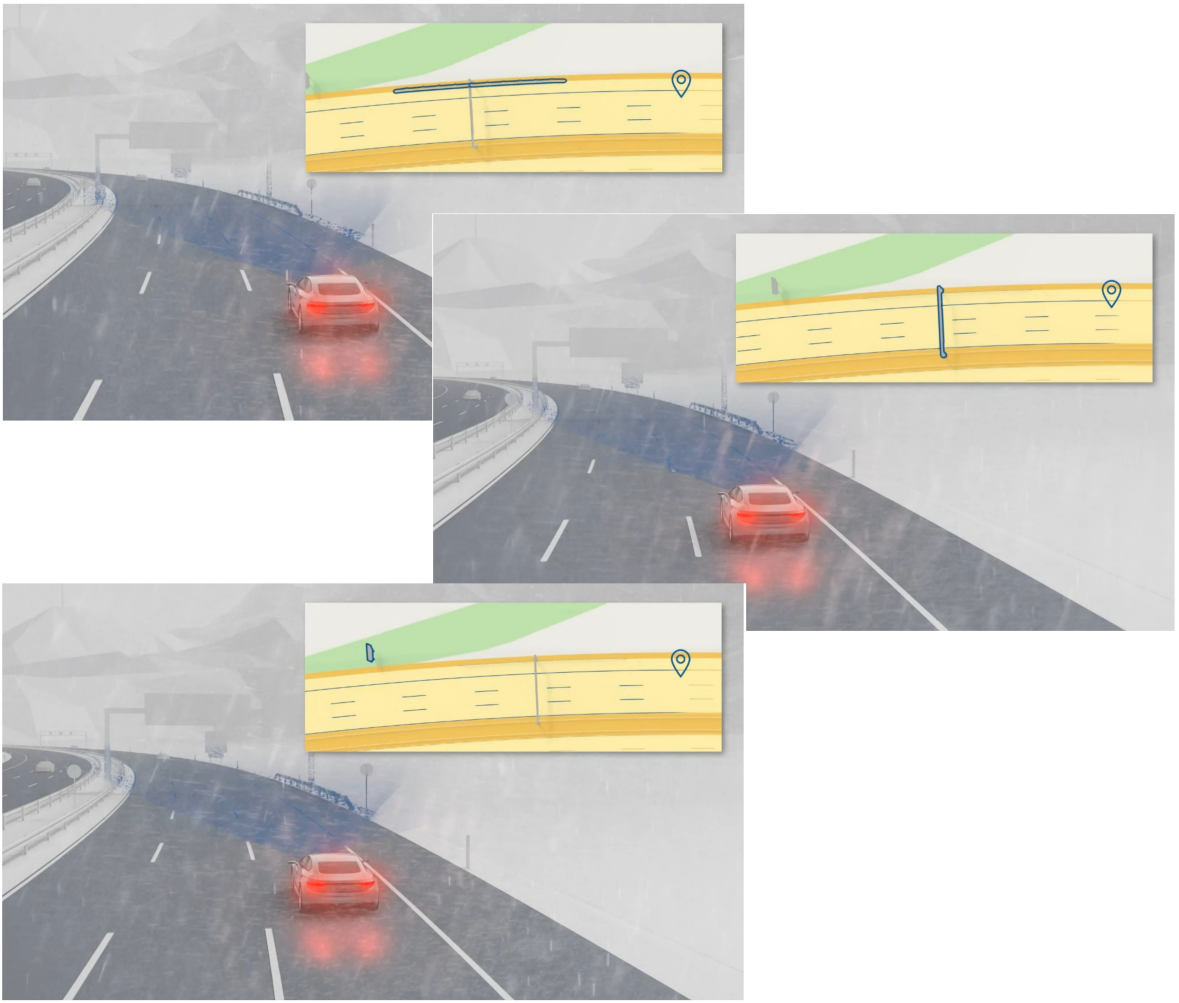
An illustration of the expected upcoming events that the autonomous car must be aware of. By having an accurate localization system, the vehicle can precisely locate the next upcoming events, so that the vehicle knows what action must be taken in the next seconds. Image source: extracted from Bosch’s video [2].

Likewise human, autonomous cars can drive more efficiently if we provide them with sufficient information that can tell them where to look and what actions to expect. An autonomous car can benefit from all this information provided it is equipped with a precise localization system. Since the navigation goal is to reach a predetermined destination location from the start position, localization can guide the vehicle to achieve the destination location in performing the navigation task. With a precise information of its position, the vehicle will be able to easily manage the maneuvers. The intersections, stop signs, curbs, lanes and turns can be handled easily because the vehicle knows where, when and what actions is expected. Figure 1 shows an illustration of the upcoming events provided by a digital map to the autonomous vehicle. With this information, the vehicle knows what actions must be taken in the next few seconds.

In order to perform complex maneuvers, an autonomous vehicle needs an accurate and robust real-time localization. Nowadays, localization has emerged as one of the crucial issues in drive-less cars development [3]. A lot of research has been done over the last decade on vehicle localization systems. The GNSS (i.e., global navigation satellite system) is one of the most widely used sensors for localizing the vehicle’s positions, such as the global positioning system (GPS). However, using only GPS solution for the localization system is not optimal to support the AV maneuvers. The accuracy of GPS often degrades due to poor satellite constellation geometry, shadowing, and multi-path propagation of satellite signals [4].

To address the GPS positioning errors, complementary systems depending on dead-reckoning sensors including accelerometer, gyroscope, and odometers have been proposed. However, the systems using these kinds of sensors have some drawbacks. Besides the expensive price issue, their drifts rapidly increase with time, and regular calibration is required [4,5]. To bridge the gaps, fusing sensors [6,7] such as visual information, digital map, and GNSS to improve localization accuracy of vehicle has emerged as a potential solution and has become one of the hottest issues in the past years [8,9,10,11].

Currently, there is much interest in the vehicle localization based on high-definition maps [2,3,4]. Digital map is used as a powerful complementary system for improving the performance of the vehicle localization. It is generated by providing sufficient information to localize vehicle’s positions relative to the map.

Common approaches have been proposed involving the combination of LIDARs, IMU, GPS and high-resolution digital maps as solutions. Fusing the measurements from vision, GPS and LIDAR have been proposed in [12,13,14]. However, the LIDAR price is still one of the big issues preventing this kind of solution from becoming wide-spread commodities [15]. Therefore, in this work, we propose a low-cost solution for lane-level localization using a vision-based system combined with map-matching method and a low-cost GPS to achieve high precision lane-level localization.

The main contributions of this paper are:

- We propose a low-cost localization system using vision-based method;
- We combine map-matching method and low-cost GPS to achieve high precision lane-level localization;
- We carry out extensive experiments in real-time and a real environment.

In general, three major steps are required: creating a reference map, finding the corresponding road segments, and positioning the vehicle on the map.

The rest of the paper is structured as follows. Related work is discussed in Section 2. Section 3 presents our proposed approach for localization. Section 4 describes the experiments and discusses the results. Section 5 draws some conclusions and future directions.

## 2. Related Works

The current localization approaches for autonomous driving can be classified into three types namely LiDAR-based, Camera-based, and Sensor fusion-based approaches.

Vehicle localization using a probabilistic map and LiDAR has been proposed in [16]. In this work, there are tree phases used for generating the map: they firstly align the overlapping returned laser beam areas, and then perform the calibration to obtain the similar response curves, and finally make a projection of the aligned calibrated trajectories into a high-resolution probabilistic map. Another LiDAR-based map-matching technique was proposed in [17]. The authors utilized the laser sensor Velodyne HDL 32-E and proposed a localization system based on particle filter. Two map-matching distance techniques have been investigated: a modified Likelihood Field distance and an adaptive standard Cosine distance. A map-matching distance between global maps and compact global map has been used to update the poses of the particles. The global map is generated offline using car’s odometry, GPS/IMU, and 3D LiDAR data and processed by GraphSLAM method. Meanwhile, the local maps are created online by employing the occupancy grid-mapping algorithm. In [18], the authors also used a Velodyne HDL-32E LIDAR and combined it with an IMU sensor to get more detailed environmental features. In this work, a two-layer LiDAR has been applied. The bottom layer contains of ground curb features, and the upper layer composed of a 2D point cloud of the vertical features. They used a combination of an a-priory map and Monte Carlo Localization (MCL) method to obtain the estimated of the vehicle’s position.

Compared to LiDAR, camera-based localization is a low-cost solution [19,20]. Stereo vision-based localization has been proposed in [21], using particle filter to have a system that can locate and navigate the vehicle on the region with the absence of lane line marking. In their work, the authors extracted lane lines using classical image processing method. They firstly preprocessed the image by applying the Gabor filter followed by the Gaussian filter in y-direction to get a gradient image. Then, based on the histogram data, the image is converted into a binary image. After that, the method fits the line marking by applying RANSAC [22] algorithm. Using the stereo cameras, the method was able to estimate the width of lane and the relative distance between the vehicle and the lane lines. Finally, the authors used particle filter to optimize these parameters. There are at least two main drawbacks of this method. Firstly, since the method uses old-fashioned line detection method, the accuracy is not optimal compared to the new line detection methods based on deep learning such as [23] and LaneNet [24] (that we exploit in this work). Secondly, the method only enables navigation in local areas because it does not use a map as a guidance when performing the navigation. Under these conditions, the vehicle can experience serious positioning errors when it comes to the global navigation especially for an autonomous vehicle. The vehicle completely blinds about what to happen in the next few seconds or minutes. The vehicle does not have enough information that can tell where to look and what actions to expect.

In [25], the authors developed video-based localization technique using point feature-based localization (PFL) and lane feature-based localization (LFL) methods to support the existing GNSS. These methods are complementing each other. PFL performs better in inner city scenarios whereas LFL is good in rural areas. Since this work is using 3D point based as features reference for map matching, it requires a lot of buffer memories to store the salient features. Such a system burdens the computational process that challenges the real-time performance. In addition to that, this system fuses two methods, the PFL and LFL. As consequence, the system can suffer from the pose update arriving out-of-sequence (OOS) because of the processing delay, and if this occurs, this would require the re-ordering and re-processing of the OOS-measurements which makes this system typically requiring a more complex architecture and slower in implementation.

Localization based on sensor fusion has been applied in [26]. The GNSS, LiDAR, and IMU sensors have been fused adaptively. The system uses Kalman filter to integrate the GNSS, SLAM and inertial navigation. The local map matching method is used to eliminate the accumulated error and to correct the positioning system. Fusing the measurements from vision, GPS and LIDAR has also been proposed in [12,13].

The high cost of LiDARs limits LiDAR-based localization for being widely used in wide-spread commodities [15]. On the other hand, the low-cost and promising performance in autonomous driving applications, vision-based approaches have shown to be appealing in solving the lane-level localization problems for autonomous driving vehicles. Therefore, we further explore vision-based technologies for developing a novel low cost lane-level localization approach.

## 3. Our Proposed Approach

**Figure 2:**
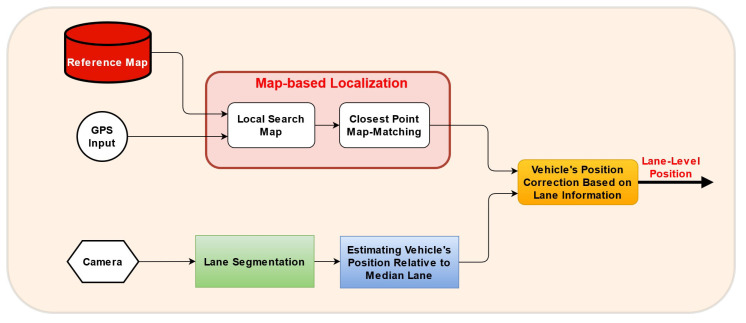
The pipeline of our proposed system.

In this work, we propose an elegant method using map-matching technique to obtain high lane-level localization accuracy. The framework of the proposed method is presented in Figure 2. Our method takes three inputs: Camera, GPS, and a Reference Map.

The GPS point constituting the trajectory is given as follows:

(1) $$p_{w}=\left(p_{x_{w}},p_{y_{w}}\right)$$

where $p_{x}$ and $p_{y}$ represent the longitude and latitude coordinates, respectively, and *w* is the current window timestamp.

The reference map positions r can be defined as follows:

(2) $$r_{j}=\left(r_{x_{j}},r_{y_{j}}\right)$$

where $r_{x}$ and $r_{y}$ represent the longitude and latitude coordinates, respectively, and *j* is the location number of the reference point.

The GPS provides the local positions and creates a local search map for an area of 25 × 25 m. The reference points of local map are selected from the reference map whose points fall inside the local area where the current GPS position is currently located. Therefore, the local reference of the window timestamp $r^{w}$ can be defined as follows:

(3) $$r_{k}^{w}=\left(r_{x_{k}},r_{y_{k}}\right)$$

where *k* is the location number of the reference point in the local map area.

The distance between these points and the current local GPS position is calculated and compared using the simple closest point algorithm.

**Figure 3:**
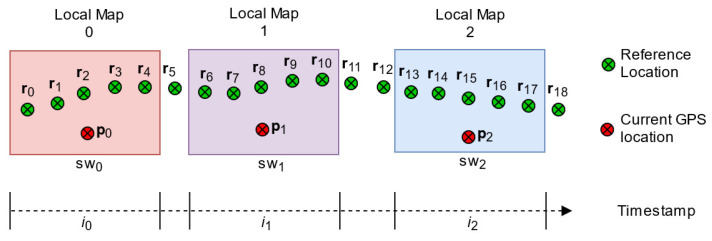
Illustration of Map-matching process using sliding window ($sw_{i}$).

Using sliding window technique, as shown in Figure 3, we search for the closest point (CP) between the position acquired from the current GPS and the positions in the reference map where the vehicle passes through it. The distances are calculated using Euclidean distance. The minimum distance is selected as the most appropriate position that is close to the vehicle. The closest point for a corresponding window is determined by the following relation:

(4) $${\mathrm{CP}}_{p_{w}}^{w}=r_{j}^{w},\;\underset{\to }{where}\;\underset{j}{\mathrm{argmin}}\left\{\sqrt{\sum_{t=1}^{2}{\left(p_{wt}-r_{jt}\right)}^{2}}\right\}$$

Simultaneously, the camera supplies the sequence of images to be processed by lane segmentation algorithm. Using lane segmentation, we have vehicle’s position relative to median lane.

### 3.1. Creating the Reference Map

**Figure 4:**
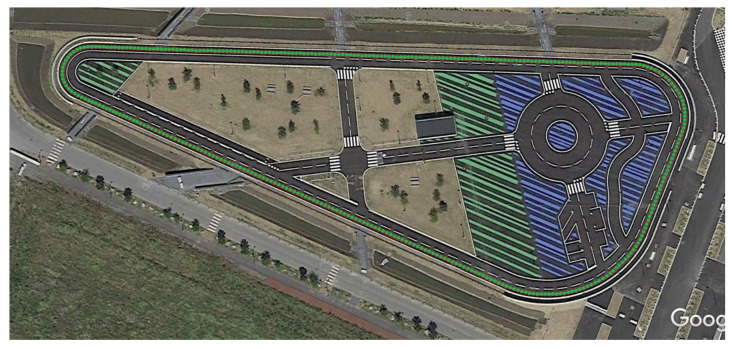
Reference map.

The reference of our map is created by leveraging Google Earth Pro. In early 2015, Google Earth Pro costed about $400, and now it is free to use [27]. We firstly created the center lane path and saved it into KML format. Then, we extracted the coordinates of this path and stored them as our reference map of the center lane. Figure 4 shows the reference map used in this work created by using Google Earth Pro.

### 3.2. Finding the Corresponding Road Segment

Roads are multi-lines. Usually, the left and right road boundaries are represented by two multi-lines. The road marks separating lanes are also shown by multi-lines. A single lane of a multi-lane feature is called road segment.

**Figure 5:**
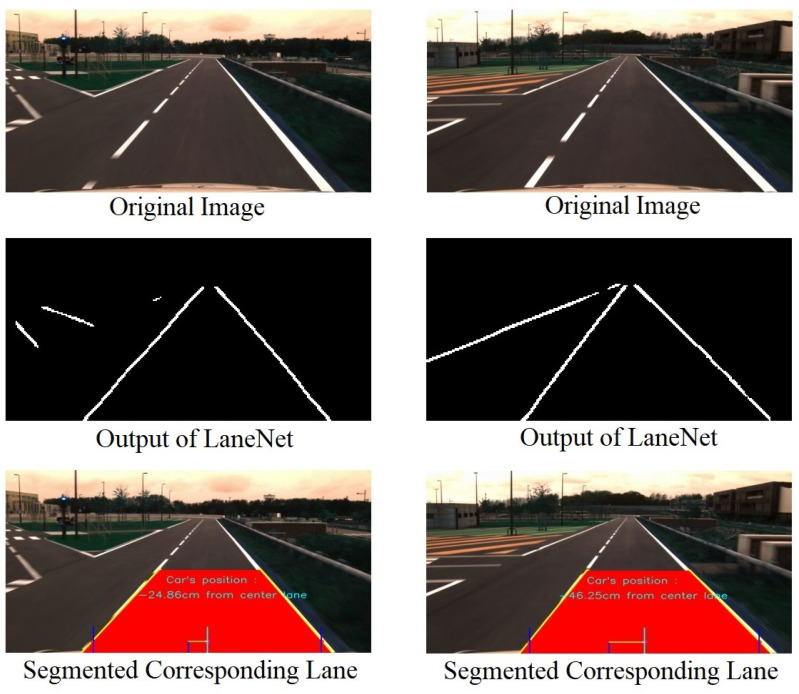
Process of finding the corresponding road segment.

One of the important steps in our approach is to localize the corresponding road segment from the input image. For this work, LaneNet [24] is employed to produce the lane segmentation. We use binary image output of the LaneNet and perform post-processing. The result of LaneNet is in form of multi-line based upon the number of road segments. In order to obtain the corresponding lane, output of LaneNet requires further processing to find the correct road segment, which is the segment where the vehicle is passing through it. To do so, we use a simple technique involving line Hough transform in order to obtain multi-line on the image. We divide the image into two sides, left and right and then we select one line on the left side and one line on the right side whose their bottom positions are closest to the bottom center of the image. These two lines are supposed to be the borders of the corresponding lane. Figure 5 shows the examples of the process of obtaining the corresponding road segment.

### 3.3. Vehicle Position on the Map

**Figure 6:**
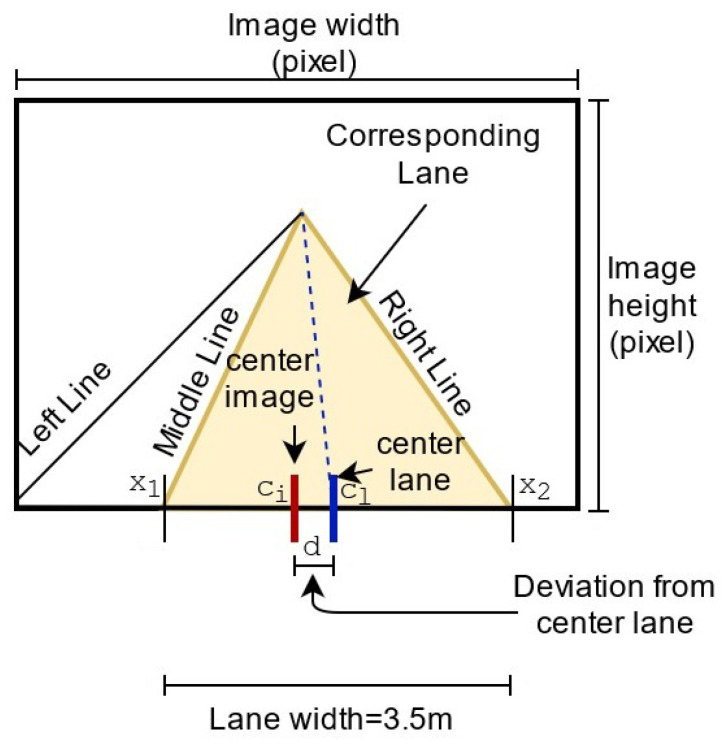
Description of how the deviation distance between the center of vehicle and middle lane is obtained.

After the implementation of the closest point map-matching method, we estimate the final position of the vehicle that is the position relative to the median lane. Figure 6 clearly illustrates the relation between the center vehicle and the median lane. The estimate distance of the center of vehicle relative to the median lane is formulated as follows:

(5) $$d_{m}=\frac{LaneWidth_{m}}{{\left(x_{2}-x_{1}\right)}_{px}}\;\;d_{px}$$

where $d_{m}$ is the estimate distance of the center vehicle relative to the median lane in *meter*, $LaneWidth_{m}$ is the width of the lane in *meter*, ${\left(x_{2}-x_{1}\right)}_{px}$ is the width of the lane in *pixel*, and $d_{px}$ is the estimate distance of the center vehicle relative to the median lane in *pixel* (Figure 6).

**Figure 7:**
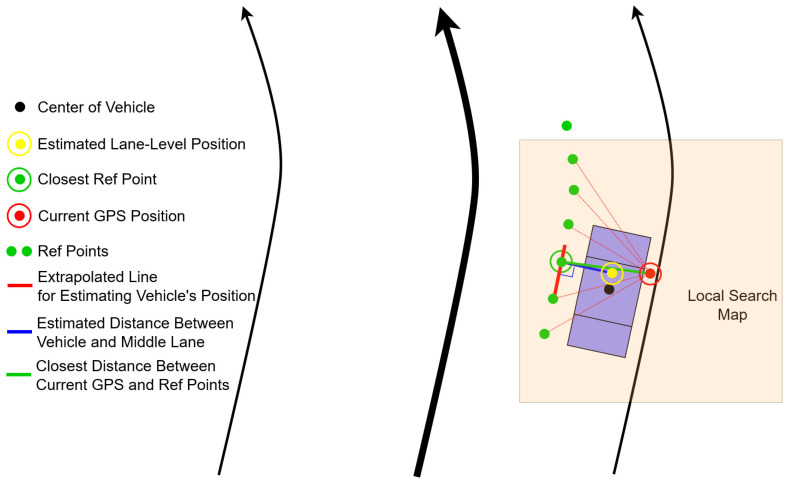
Illustration of estimating vehicle’s position based on map-matching.

Using the estimated distance between the center of vehicle relative to the center lane, the lane-level localization is performed as shown Figure 7.

## 4. Experiments and Results

**Figure 8:**
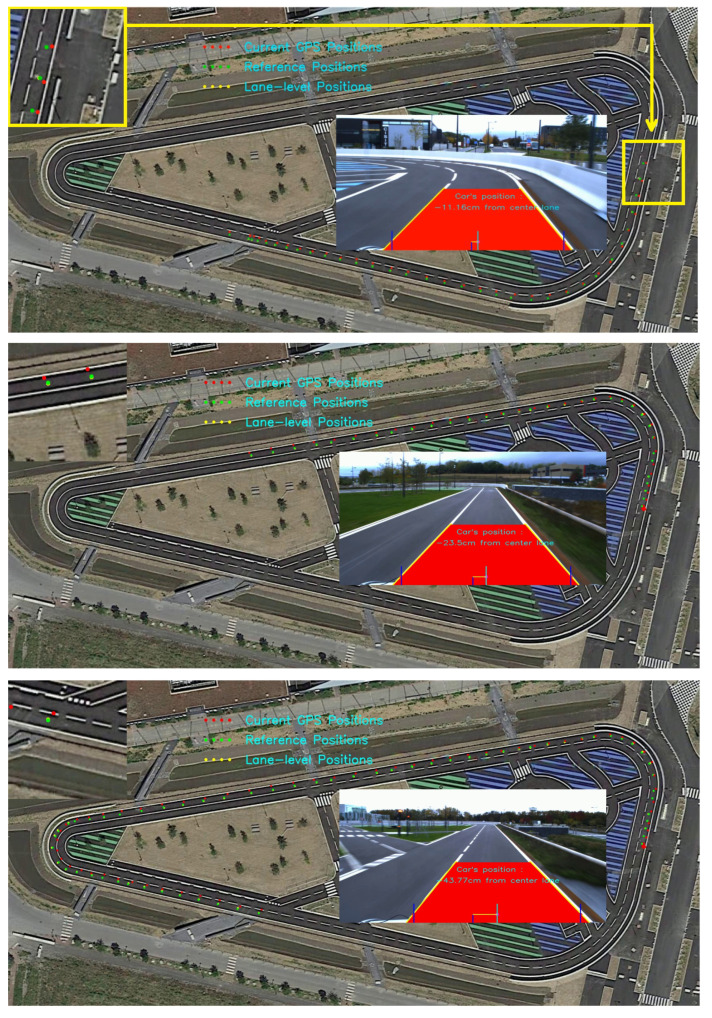
Qualitative localization results of the proposed method.

In this work, we carried out all our experiments on our test track under natural lighting conditions. Our proposed approach was tested in a track whose length is 850 m. The track is composed of two lanes of width of 3.5 m each. The current position of the vehicle is measured using a low cost GPS receiver mounted on the top of a testing car. When conducting the experiments, we do not have a high precision GPS to be used as a ground-truth data. This way we measured the deviation of distance from the middle lane to the center of the vehicle. By assuming that the vehicle should most of the time be in the middle lane, we measured vehicle’s position relative to the median lane as our performance metric. Table 1 shows the obtained results of the comparison between the GPS positioning and the estimated position obtained using our proposed method. The qualitative results of the experiments can be seen in Figure 8.

Based on Table 1, the measured GPS position has higher variance and higher mean values than the estimated position. This indicates that the GPS measures are not accurate enough to localize the position of the car. Compared to the measured GPS, our proposed estimated position has smaller mean and variance values. This indicates that the proposed method works better than the only GPS approach. This also indicates that most of the times the vehicle follows the median lane.

**Figure 9:**
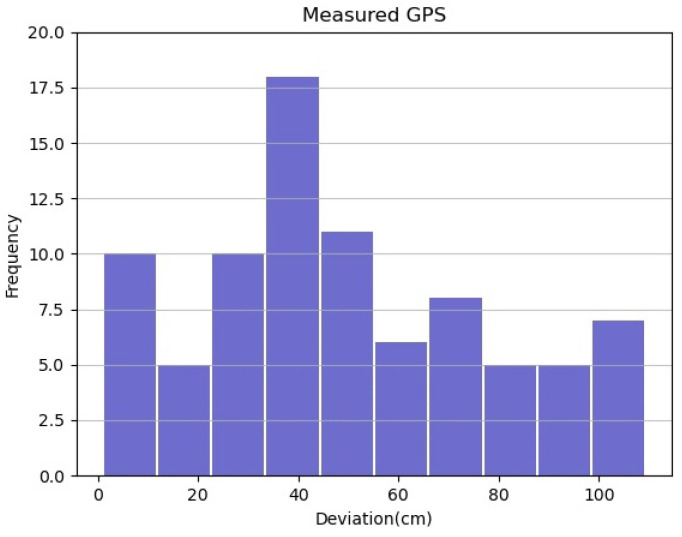
Histogram of deviation between center vehicle and median lane using only GPS.

**Figure 10:**
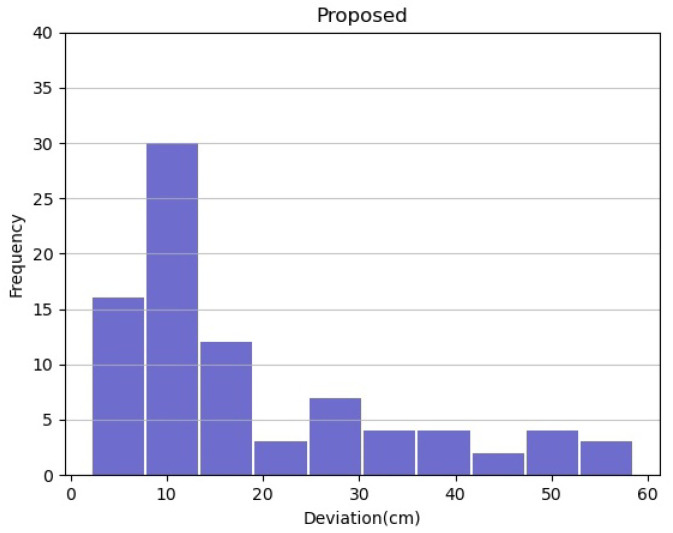
Histogram of deviation between center vehicle and median lane using the proposed method.

The comparative results can also be seen in Figure 9 and Figure 10. It is clear that the proposed method localizes the vehicle more accurately than using only GPS. The proposed method has smaller deviations, which are less than 60 cm, and mostly in range of 5 to 15 cm, compared to the only use GPS, which are up to 110 cm, and most of them in range of 30 to 35 cm.

**Figure 11:**
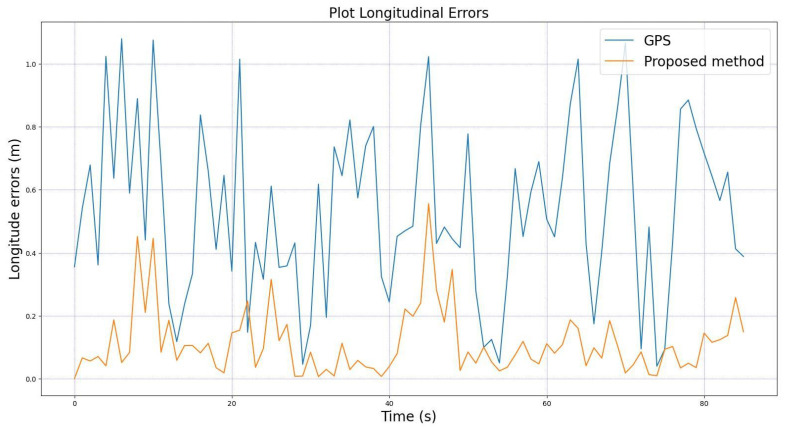
Comparison of longitudinal errors between the GPS and the proposed method.

**Figure 12:**
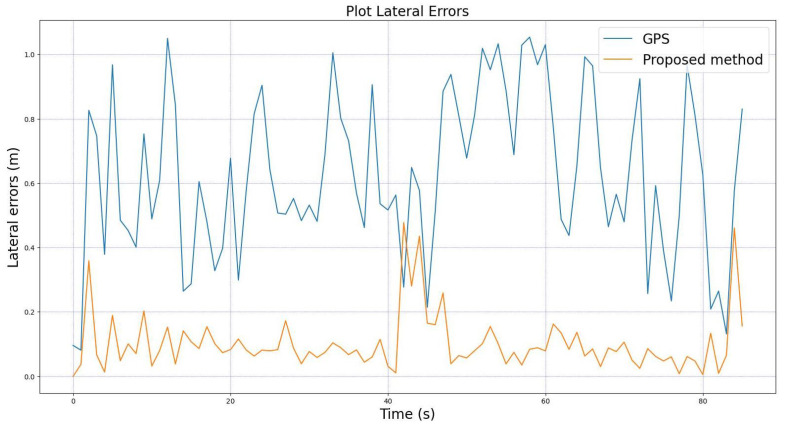
Comparison of lateral errors between the GPS and the proposed method.

For comprehensive analysis, we also provide the comparisons between the longitudinal and lateral errors of the GPS and from the proposed method relative to the median lane. These comparisons are shown in Figure 11 and Figure 12. The figures clearly show that our proposed method has very small errors compared to the GPS for both longitudinal and lateral errors. These results confirm once again that our proposed method yields a better performance in localizing the vehicle compared to the systems using only GPS.

## 5. Conclusions

In this paper, we proposed a low-cost solution for a localization system using vision-based system combined with map-matching method and low cost GPS to achieve high precision lane-level localization. In general, three major steps are required: creating reference map, finding the corresponding road segment, and positioning vehicle on the map. Our proposed approach was applied for real-time measurement of vehicle’s position relative to median lane. The obtained results showed better performance compared to measured GPS position.

Please note the preliminary results of this work were published at AISC2021 workshop. This extended version provides more figures and more explanations and interpretations of the experimental results. To support the principle of reproducible research and fair comparison, we plan to release (GitHub, under construction) all the code and material for the research community.

## Figures and Tables

**Table 1:** Performance comparison between the GPS and the proposed method.

	Dev. in Measured Position	Dev. in Estimated Position
Mean (cm)	49.30	29.52
Standard deviation (cm)	18.65	14.53

## Data Availability

Not applicable.

## References

[1] Petrovskaya A., Thrun S. (2009). Model based vehicle detection and tracking for autonomous urban driving. Auton. Robot..

[2] Bosch Localization for Automated Driving. https://www.bosch-mobility-solutions.com/en/solutions/automated-driving/localization-for-automated-driving/.

[3] Kim D., Kim B., Chung T., Yi K. (2017). Lane-Level Localization Using an AVM Camera for an Automated Driving Vehicle in Urban Environments. IEEE/ASME Trans. Mechatron..

[4] Cui D., Xue J., Zheng N. (2016). Real-Time Global Localization of Robotic Cars in Lane Level via Lane Marking Detection and Shape Registration. IEEE Trans. Intell. Transp. Syst..

[5] Abbott H., Powell D. (1999). Land-vehicle navigation using GPS. Proc. IEEE.

[6] Li T., Qian Y., de La Fortelle A., Chan C.-Y., Wang C. (2020). Lane-level localization system using surround-view cameras adaptive to different driving conditions. Int. J. Adv. Robot..

[7] Hosseinyalamdary S., Peter M. Lane level localization; using images and HD maps to mitogate the latteral error. Proceedings of the International Archives of the Photogrammetry, Remote Sensing & Spatial Information Sciences.

[8] Dixiao C., Jianru X., Shaoyi D., Nanning Z. Real-time global localization of intelligent road vehicles in lane-level via lane marking detection and shape registration. Proceedings of the 2014 IEEE/RSJ International Conference on Intelligent Robots and Systems.

[9] Vu A., Ramanandan A., Chen A., Farrell J., Barth M. (2012). Real-time computer vision/DGPS-aided inertial navigation system for lane-level vehicle navigation. IEEE Trans. Intell. Transp. Syst..

[10] Schreiber M., Knoppel C., Franke U. LaneLoc: Lane marking based localization using highly accurate maps. Proceedings of the 2013 IEEE Intelligent Vehicles Symposium (IV).

[11] Tao Z., Bonnifait P., Fremont V., Ibanez-Guzman J. Lane marking aided vehicle localization. Proceedings of the 16th International IEEE Conference on Intelligent Transportation Systems (ITSC 2013).

[12] Rose C., Britt J., Allen J., Bevly D. (2014). An integrated vehicle navigation system utilizing lane-detection and lateral position estimation systems in difficult environments for gps. IEEE Trans. Intell. Transp. Syst..

[13] Li Q., Chen L., Li M., Shaw S.-L., Nuchter A. (2014). A sensor-fusion drivable-region and lane-detection system for autonomous vehicle navigation in challenging road scenarios. IEEE Trans. Veh. Technol..

[14] Ballardini A.L., Fontana S., Cattaneo D., Matteucci M., Sorrenti D.G. (2021). Vehicle Localization Using 3D Building Models and Point Cloud Matching. Sensors.

[15] Hillel A.B., Lerner R., Levi D., Raz G. (2014). Recent progress in road and lane detection: A survey. Mach. Vis. Appl..

[16] Levinson J., Thrun S. Robust vehicle localization in urban environments using probabilistic maps. Proceedings of the 2010 IEEE International Conference on Robotics and Automation.

[17] De Paula Veronese L., Guivant J., Auat Cheein F.A., Oliveira-Santos T., Mutz F., de Aguiar E., Badue C., De Souza A.F. A light-weight yet accurate localization system for autonomous cars in large-scale and complex environments. Proceedings of the 2016 IEEE 19th International Conference on Intelligent Transportation Systems (ITSC).

[18] Wang Z., Fang J., Dai X., Zhang H., Vlacic L. (2020). Intelligent vehicle self-localization based on double-layer features and multilayer lidar. IEEE Trans. Intell. Veh..

[19] Jo K., Jo Y., Suhr J.K., Jung H.G., Sunwoo M. (2015). Precise localization of an autonomous car based on probabilistic noise models of road surface marker features using multiple cameras. IEEE Trans. Intell. Transp. Syst..

[20] Kang J.M., Yoon T.S., Kim E., Park J.B. (2020). Lane-level map-matching method for vehicle localization using GPS and camera on a high-definition map. Sensors.

[21] Wen L., Jo K. Vehicle localization and navigation on region with disappeared lane line marking. Proceedings of the 2016 IEEE/SICE International Symposium on System Integration (SII).

[22] Fischler M.A., Bolles R.C. (1981). Random Sample Consensus: A Paradigm for Model Fitting with Applications to Image Analysis and Automated Cartography. Commun. ACM.

[23] Bai M., Mattyus G., Homayounfar N., Wang S., Lakshmikanth S.K., Urtasun R. Deep multi-sensor lane detection. Proceedings of the 2018 IEEE/RSJ International Conference on Intelligent Robots and Systems (IROS).

[24] MaybeShewill-CV (2020). Lanenet-Lane-Detection. GitHub Repository. https://github.com/MaybeShewill-CV/lanenet-lane-detection.

[25] Ziegler J., Lategahn H., Schreiber M., Keller C.G., Knöppel C., Hipp J., Haueis M., Stiller C. Video based localization for bertha. Proceedings of the 2014 IEEE Intelligent Vehicles Symposium Proceedings.

[26] Xia Z., Tang S. Robust self-localization system based on multi-sensor information fusion in city environments. Proceedings of the 2019 International Conference on Information Technology and Computer Application (ITCA).

[27] The University of Waterloo Google Earth Pro: A Tutorial. https://uwaterloo.ca/library/geospatial/sites/ca.library.geospatial/files/uploads/files/google_earth_2016.pdf.

